# From fasting to fat reshaping: exploring the molecular pathways of intermittent fasting-induced adipose tissue remodeling

**DOI:** 10.3389/jpps.2024.13062

**Published:** 2024-07-22

**Authors:** Nathaniel Vo, Qiwei Zhang, Hoon-Ki Sung

**Affiliations:** ^1^ Translational Medicine Program, The Hospital for Sick Children, Toronto, ON, Canada; ^2^ Department of Laboratory Medicine and Pathobiology, University of Toronto, Toronto, ON, Canada

**Keywords:** obesity, intermittent fasting, adipose tissue remodeling, angiogenesis, sympathetic innervation

## Abstract

Obesity, characterised by excessive fat accumulation, is a complex chronic condition that results from dysfunctional adipose tissue expansion due to prolonged calorie surplus. This leads to rapid adipocyte enlargement that exceeds the support capacity of the surrounding neurovascular network, resulting in increased hypoxia, inflammation, and insulin resistance. Intermittent fasting (IF), a dietary regimen that cycles between periods of fasting and eating, has emerged as an effective strategy to combat obesity and improve metabolic homeostasis by promoting healthy adipose tissue remodeling. However, the precise molecular and cellular mechanisms behind the metabolic improvements and remodeling of white adipose tissue (WAT) driven by IF remain elusive. This review aims to summarise and discuss the relationship between IF and adipose tissue remodeling and explore the potential mechanisms through which IF induces alterations in WAT. This includes several key structural changes, including angiogenesis and sympathetic innervation of WAT. We will also discuss the involvement of key signalling pathways, such as PI3K, SIRT, mTOR, and AMPK, which potentially play a crucial role in IF-mediated metabolic adaptations.

## Introduction

Obesity is a growing epidemic, impacting individuals and societies on a global scale. It is a complex chronic disease marked by the accumulation of excess body fat, or adiposity, which negatively affects an individual’s health [1]. The impact of obesity extends beyond increased body weight and is linked with a myriad of cardiometabolic diseases, such as type 2 diabetes (T2D), hypertension, and atherosclerosis, in addition to respiratory diseases and certain types of cancer [2, 3]. White adipose tissue (WAT) is a critical endocrine organ implicated in the progression of obesity. WAT is associated with an extensive neurovascular network and contains a heterogeneous population of cells, including mature adipocytes, adipose stem and progenitor cells (ASPCs) and vascular endothelial and immune cells [4]. These interactions contribute to the overall function of WAT in regulating whole-body metabolism and systemic homeostasis.

**FIGURE 1:**
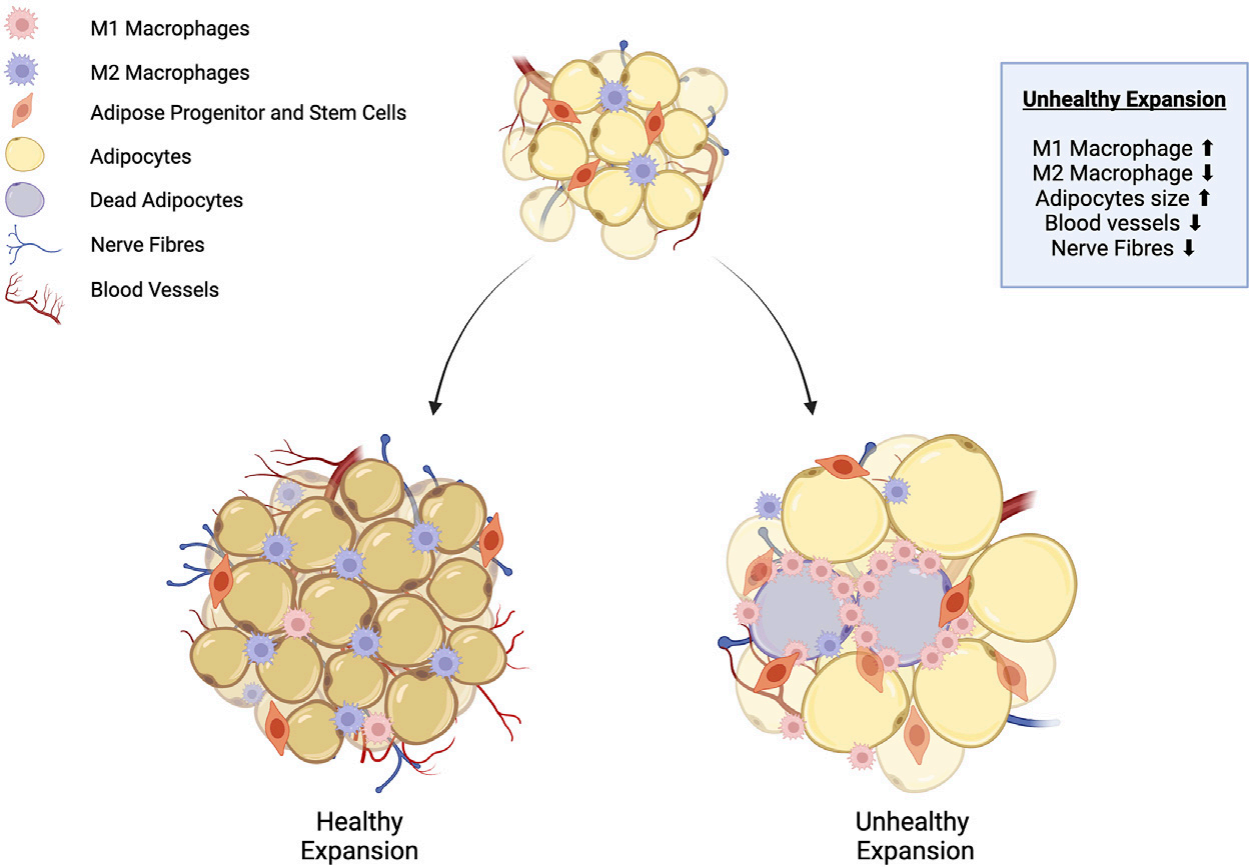
Adipose tissue remodeling: healthy vs. unhealthy WAT expansion. Healthy white adipose tissue (WAT) expansion is characterised by the proliferation of adipose stem and progenitor cells (ASPCs) into numerous small adipocytes. At the same time, anti-inflammatory M2 macrophages are recruited into the adipose tissue, and expansion is supported by the growth of the surrounding neurovascular architecture. Conversely, unhealthy WAT expansion is characterised by impaired differentiation of ASPCs into mature adipocytes. Lipids accumulate within existing adipocytes, causing them to progressively enlarge. Some adipocytes undergo apoptosis due to inadequate nutrient supply from blood vessels, which triggers the recruitment of pro-inflammatory M1 macrophages, leading to increased inflammation. Adapted from Choe et al. [5], licensed under CC BY 4.0. Created with BioRender.com.

Adipose tissue remodeling is a biological process that involves changes in the morphology, cellular composition, and function of adipose tissue in response to physiological or pathological stimuli (Figure 1) [6]. A central component of the remodeling process is WAT expansion, which is characterised by two physiological processes: adipocyte hypertrophy, which is the increase in cell size, and hyperplasia, which leads to an increase in adipocyte cell number through adipogenesis [5]. The process of adipogenesis involves the differentiation of ASPCs into mature adipocytes and is controlled by a subset of regulatory signalling pathways [7].

It is also accompanied by the development of a supportive neurovascular architecture through signalling mechanisms such as the canonical vascular endothelial growth factor (VEGF)/VEGF Receptor 2 (VEGFR2). VEGF signalling is known to regulate angiogenesis, the growth of blood vessels, sympathetic innervation, and the extension and branching of sympathetic nerve fibres within adipose tissue [8]. Angiogenesis is critical for the delivery of essential nutrients, the removal of metabolic waste, the transport of adipokines, and the mobilisation of free fatty acids released during lipolysis [9]. Sympathetic innervation plays a significant role in the regulation of lipolysis, adipocyte proliferation, and thermogenesis [10–12].

A chronic imbalance between energy intake and expenditure can give rise to dysfunctional adipose tissue expansion, where adipocyte hypertrophy predominates and exceeds the capacity of the surrounding neurovascular network. This imbalance can result in hypoxia, triggering a cascade of inflammatory responses that ultimately contribute to the development of insulin resistance [13–15]. Thus, this highlights the considerable importance of adipose tissue remodeling in obesity, positioning it as a key target for therapeutic intervention.

Current strategies for managing obesity include dietary regimens, physical exercise, pharmacotherapy, and, in certain cases, bariatric surgery [16–19]. Among dietary approaches, calorie restriction (CR), which involves a decrease in total calories consumed, has been extensively studied and clinically implemented. CR is linked to numerous health benefits, including improvements in cardiovascular and metabolic health, cognitive function, and longevity [20–23]. However, maintaining weight loss through long-term adherence to a calorie-restricted regimen can be difficult, often resulting in poor compliance and subsequent weight regain once the regimen is discontinued [24, 25].

Given these challenges, intermittent fasting (IF) has emerged as a promising dietary strategy over the past decade. Unlike CR, it involves cycles of defined fasting and eating periods without necessarily limiting the number of calories an individual consumes. Instead, IF restricts food consumption to certain hours of the day or specific days of the week. This regimen yields metabolic benefits similar to CR, including reduced body mass, improved adipose tissue inflammation and insulin sensitivity, and improved cardiometabolic health without reducing calorie intake [26–31]. Directly comparing the metabolic improvements between CR and IF requires additional studies, which are currently limited and ongoing [32–34].

IF encompasses a diverse range of dietary protocols that vary in the duration, frequency, and extent of calorie reduction during fasting periods. One notable strategy is alternate day fasting (ADF), which can be further categorised as complete ADF, involving zero calorie intake on fasting days, or modified ADF, which allows approximately 25% of an individual’s daily calorie intake to be consumed during fasting periods. This adaptation mitigates some of the challenges associated with ADF and improves sustainability and adherence [35].

Periodic Fasting (PF) represents another form of IF, and it involves extended fasting periods or significantly reduced calorie intake interspersed with normal eating periods. Unlike ADF, PF typically involves longer fasting intervals of 2 days or more [29, 36]. The 5:2 IF regimen, one of the most recognised and popularised, allows unrestricted eating 5 days a week, with reduced calorie consumption on either two consecutive or non-consecutive days [37].

Time-restricted feeding (TRF) is a protocol that restricts food intake to a specific 8-h daily window, outside of which only water is allowed [38]. Recent clinical studies have highlighted that early TRF, with the window starting at 06:00 AM, further improves metabolic parameters such as fasting glucose and insulin sensitivity [39, 40]. These benefits are mediated, in part, by the synchronicity of TRF with our circadian rhythm. Ramadan fasting (RF) is a unique form of TRF practised by Muslims during the month of Ramadan, which involves restricted eating and drinking from dawn (Suhur) to sunset (Iftar) [41]. The duration of fasting depends on the time of year and geographical location. A recent meta-analysis by Fernando et al highlighted reductions in body weight and fat mass following RF, along with improvements in several metabolic markers such as fasting glucose and low-density lipoprotein levels [42, 43].

Despite the growing popularity and adoption of various IF protocols, the underlying biological mechanisms that facilitate these benefits remain to be elucidated. Existing research has demonstrated that IF promotes a range of metabolic adaptations, including enhanced lipolysis, β-oxidation, gut microbiota changes, and increased autophagy [44–48]. IF also significantly influences the browning of WAT, leading to the formation of beige adipocytes, which contain smaller lipid droplets and a higher mitochondrial density [26, 28, 49, 50]. This browning effect facilitates heat dissipation through respiratory uncoupling via uncoupling protein 1 (UCP1), a process known as WAT thermogenesis, which is critical in regulating energy expenditure and metabolic homeostasis [26, 28, 49, 50]. These processes contribute to the metabolic benefits observed with IF, including improved insulin sensitivity and glucose tolerance.

However, significant gaps persist in our understanding of the specific molecular pathways involved and the differential effects of IF on various tissues and organs. In this review, we aim to explore the signalling pathways, with a focus on adipose tissue that is influenced by IF and its downstream effects on adipose tissue remodeling. We also aim to identify key areas for future research to improve our current knowledge of the molecular targets that drive these metabolic adaptations. This understanding will set the stage for the development of pharmacological interventions that can mimic the beneficial effects of IF, potentially offering new strategies for the management of obesity.

## Intermittent fasting and associated signalling pathways

### Phosphatidylinositol 3-kinase (PI3K) and protein kinase B (Akt) pathways

The PI3K/Akt signalling pathway coordinates various anabolic processes that are essential for maintaining cell growth and proliferation, glucose and lipid metabolism, and autophagy. This pathway is crucial for insulin signal transduction and exerts beneficial effects on glucose homeostasis through the regulation of FOXO1 and GSK3β, mitochondrial biogenesis via mTORC1, and lipogenesis through the regulation of PPARγ and SREBP1-c [50].

Activation of PI3K/Akt signalling in adipocytes has been linked to improved insulin sensitivity and glucose tolerance, while also promoting lipogenesis over lipolysis *in vitro* and *in vivo* [51–54]. During the refeeding phase of IF in mice, there is an upregulation of CDC-like kinase 2 (CLK2) in brown adipose tissue, which contributes to the increased energy expenditure observed with IF. This upregulation may be regulated by insulin and PI3K signalling since treatment with a PI3K inhibitor has been shown to ameliorate the upregulation of CLK2 [55].

Although direct evidence connecting IF with PI3K/Akt signalling in WAT is still lacking, CR in mice has been shown to improve insulin sensitivity and promote lipid metabolism through Akt activation, particularly in the liver [56]. Catalpol, a naturally derived drug, has been shown to alleviate hepatic insulin resistance and improve glucose homeostasis in mice by stimulating AMP-activated protein kinase (AMPK) and PI3K/Akt signalling. Knockdown of AMPK in HepG2 cells was observed to prevent Akt phosphorylation and activation induced by Catalpol [57].

Furthermore, in a mouse model of diet-induced obesity (DIO), a 5-week ADF regimen mitigated obesity-induced remodeling of the atria. It also showed significant improvements in glucose tolerance and insulin sensitivity via SIRT3 and its downstream activation of AMPK and Akt [58]. Additionally, by modelling chronic myocardial ischaemia in rats, Katare et al demonstrated that long-term IF led to a substantial improvement in survival rates through activation of the BDNF/VEGF/PI3K signalling cascade in the heart, with a significant upregulation of phospho-Akt [59].

Conversely, the downregulation of PI3K/Akt signalling is also highlighted in specific circumstances. Butein, a phytochemical, can induce WAT beiging in DIO mice through inhibition of PI3K and downstream Akt signalling, leading to activation of PRDM4, a regulator of energy expenditure and thermogenesis [60]. A study of ADF in rats showed improvement in age-associated hypertrophy via downregulation of PI3K/Akt signalling in the heart’s left ventricle [61]. Moreover, PI3K/Akt activation was also inhibited in the hippocampal region following IF, activating GSK3β and promoting neuronal differentiation in a mouse model of Alzheimer’s disease [62]. Similarly, the hypothalamus showed reduced activation of PI3K/Akt/mTOR signalling following short-term fasting in rats [63].

IF facilitates the metabolic switch between catabolic and anabolic states in response to fasting and refeeding. This may result in the inhibition or activation of PI3K/Akt signalling. These findings illustrate the complex and context-dependent relationship between IF and PI3K/Akt signalling in various tissues, including adipose tissue, liver, heart, and brain. This dual regulation may explain how IF promotes insulin sensitivity while also stimulating catabolic processes such as WAT lipolysis and thermogenesis. Further research is essential to unravel the precise mechanisms of IF-induced modulation of the PI3K/Akt pathway in WAT.

### Sirtuins (SIRTs) pathway

SIRTs are a family of nicotine adenine dinucleotide (NAD)+-dependent deacetylases that regulate numerous cellular processes, including metabolism, ageing, and oxidative stress [64, 65]. SIRTs catalyse the removal of acyl groups from target proteins and function as metabolic regulators in response to changes in NAD+ levels. The SIRT family consists of seven members, SIRT1 through SIRT7, each residing in specific subcellular compartments [64].

In individuals with obesity, SIRT6 expression in WAT was found to be significantly reduced [66]. Mice lacking SIRT6 in adipocytes, when subjected to a high-fat diet (HFD), manifested exacerbated insulin resistance and inflammation [66]. Similarly, adipocyte-specific SIRT6 knockout mice undergoing IF failed to show improvements in glucose homeostasis and insulin sensitivity. The absence of SIRT6 also led to reduced adipose browning and lower energy expenditure, suggesting that SIRT6 is a critical mediator of the metabolic improvements induced by IF [67].

Moreover, CR in rats and fasting in humans have been shown to upregulate SIRT1 expression in WAT [68, 69]. SIRT1 promotes WAT browning and increases energy expenditure by deacetylating PPARγ [70]. However, Boutant et al revealed that while SIRT1 overexpression leads to similar improvements in insulin sensitivity, it cannot replicate the effects of ADF on WAT metabolism, including increased mitochondrial respiration and distinct transcriptional alterations in epididymal WAT [71].

SIRT7 can also significantly affect lipid metabolism and thermogenesis [72, 73]. Yoshizawa et al found that whole-body and brown adipose tissue-specific SIRT7 knockout mice display augmented body temperature and energy expenditure along with increased UCP1 expression [73]. Furthermore, Tang et al demonstrated that IF can promote SIRT7 stability by regulating AMPK activity, thereby activating the GSK3β-SIRT7 axis in the context of enhancing the anti-tumour effects of chemotherapy [74]. These findings underscore the potential role of SIRTs in WAT metabolism and remodeling. Nevertheless, additional research is required to delineate the contributions of SIRT7 and other SIRTs within WAT.

### Mammalian target of the rapamycin (mTOR) pathway

The mTOR signalling pathway is a critical regulator of autophagy, among its numerous roles in influencing growth, proliferation, glucose, and lipid metabolism [75]. Two functional protein complexes are involved in the regulation of mTOR signalling: mTOR complex 1 (mTORC1) and mTOR complex 2 (mTORC2). mTORC1 is known to directly phosphorylate and inhibit the activity of Unc-51-like kinase 1 (ULK1), thus negatively regulating autophagy [75].

The mTOR pathway is activated under conditions of excess calorie intake. This activation facilitates *de novo* lipogenesis by activating sterol regulatory element-binding protein 1 (SREBP1) while concurrently suppressing lipolysis by downregulating the expression of lipolytic enzymes such as adipose triglyceride lipase (ATGL) and hormone-sensitive lipase (HSL) [76, 77]. Moreover, activation of mTORC1 is associated with increased adipogenesis, which supports the storage of excess lipids [78]. Knock out of *raptor*, a component of mTORC1, in adipocytes results in increased insulin sensitivity, reduced fat mass, and elevated energy expenditure in mice. This is achieved through the beiging of WAT, as evidenced by the upregulation of UCP1 and other browning markers [79]. These observed metabolic benefits are similar to those promoted by IF, highlighting the role of mTOR signalling in the regulation of WAT function and metabolism.

The inhibitory effects of IF on mTOR signalling in WAT have been largely unexplored. However, previous studies have shown the beneficial effects of IF and mTOR signalling in the heart. This is mediated through the intermittent activation of the transcription factor EB (TFEB), a positive regulator of autophagy [80]. Ma et al showed that IF can reduce the activation of mTOR, a negative regulator of TFEB, thereby stimulating TFEB and facilitating protective autophagic effects in a mouse model of desmin-related cardiomyopathy [81–83]. Notably, the phytochemical Acteoside can stimulate beige adipocyte formation *in vitro* via the mTORC1-TFEB signalling pathway, where upregulation of browning markers such as PGC-1α and UCP1 has been observed [84]. Whether IF can mediate autophagy and other positive effects on WAT remodeling via mTOR-TFEB signalling remains unknown and warrants further investigation.

### AMPK as the upstream regulator of PI3K/Akt, SIRTs, and mTOR signalling

AMPK is a widely expressed serine/threonine kinase that functions as a metabolic switch, responding to changes in adenosine monophosphate (AMP)/adenosine triphosphate (ATP) levels within the cell. AMPK functions to restore energy balance by favouring catabolic processes that generate ATP while suppressing anabolic pathways [85]. AMPK is activated in response to various metabolic challenges, including exercise, cold exposure, and fasting [86–88]. Fasting can activate AMPK directly or indirectly, through the increased production of metabolic hormones such as catecholamines and adiponectin [86, 89, 90].

AMPK can activate PI3K/Akt signalling as demonstrated *in vitro* by treating differentiated 3T3-L1 adipocytes with AICAR, an AMPK agonist [91]. Impairments in AMPK and PI3K/Akt signalling, observed in obesity and T2D, may contribute to insulin resistance in humans [92, 93]. However, the relationship between AMPK and PI3K/Akt is complex and context-dependent, as activation of AMPK favours catabolic processes. Fasting-induced activation of AMPK can, in contrast, directly inhibit mTOR, leading to increased autophagy via ULK1 activation. This process also promotes catabolic pathways such as lipolysis and β-oxidation while suppressing adipogenesis and lipogenesis [94].

IF triggers significant fluctuations in cellular energy levels, providing a repeated stimulus that may give rise to the remodeling of WAT via activation of AMPK. Several studies have highlighted AMPK as a critical factor in regulating glucose and lipid metabolism, and driving thermogenesis via WAT browning [90, 95, 96]. Conversely, impairment in AMPK activity is associated with various metabolic disorders, including obesity and insulin resistance, type 2 diabetes and fatty liver disease [88, 90]. Although IF has recently been demonstrated to promote AMPK activation and remodeling in the hearts of rats, its role in facilitating IF-induced remodeling of WAT remains elusive [97].

## Intermittent fasting and angiogenic remodeling

IF, similar to cold exposure and exercise, is a potent stimulus for promoting adipose tissue thermogenesis via the beiging of WAT [26, 49]. This is favourable in the context of obesity, where increasing energy expenditure may be one non-pharmacological approach to its management. IF is known to induce brown fat-like changes via adipose angiogenic remodeling. 2:1 IF (48-hour feeding, 24-hour fasting) under high-fat diet (HFD) conditions resulted in improved glucose tolerance, insulin sensitivity, and increased energy expenditure. These metabolic benefits are dependent on an IF-stimulated increase in VEGF-A expression, which leads to visceral WAT browning (upregulation of *Adrb3*, *Ppargc1a*, *Cidea*, *Ucp1*) via M2-like macrophage polarisation (upregulation of *Clec10a*, *Il10*, *Ym1*) [26]. IF can mediate browning in perigonadal and inguinal WAT (pWAT and iWAT, respectively), and higher energy expenditure was observed under both normal-chow and HFD-IF regimens [28].

**FIGURE 2:**
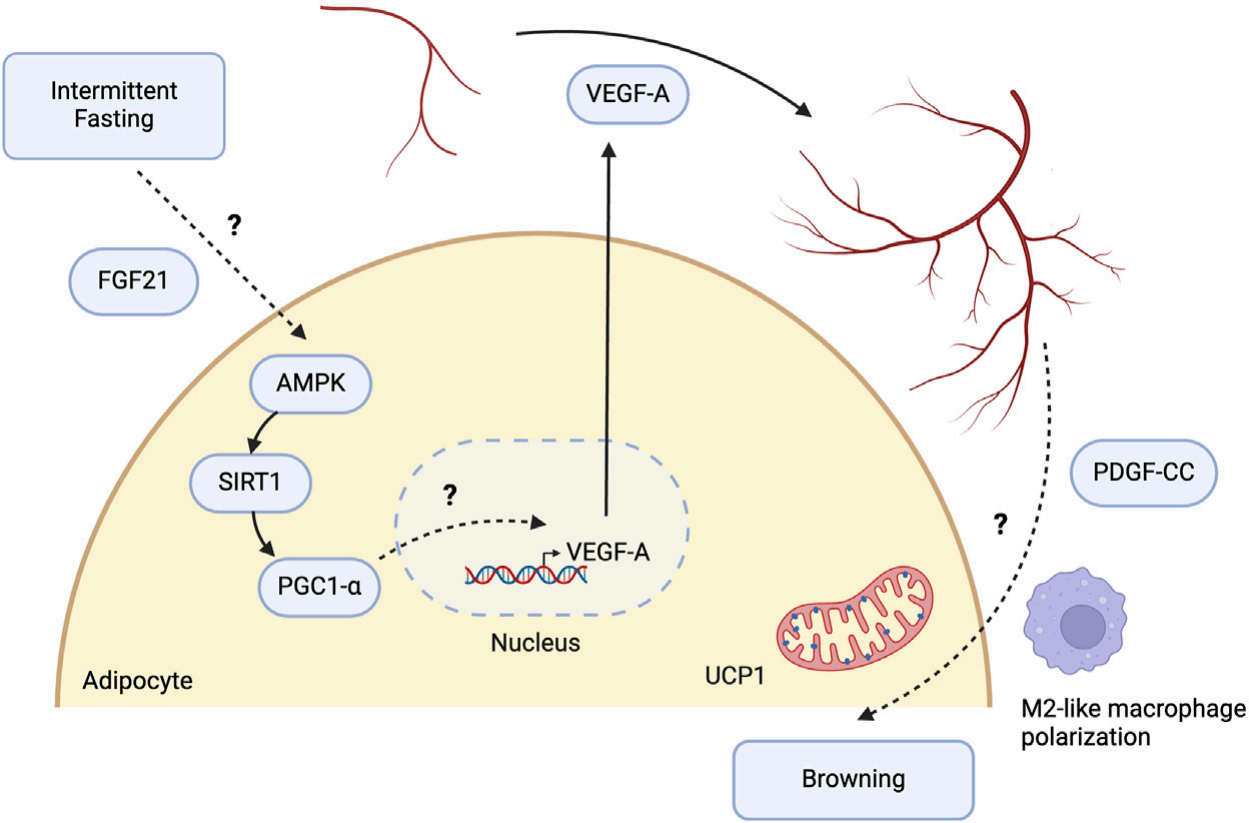
Potential molecular pathways involved in intermittent fasting-induced angiogenic remodeling of white adipose tissue. Intermittent fasting (IF) induces the expression of vascular endothelial growth factor A (VEGF-A) in white adipose tissue (WAT), promoting angiogenesis and browning. Liver-derived fibroblast growth factor 21 (FGF21) is indispensable for IF-mediated angiogenesis and browning in WAT. FGF21 upregulates the AMPK-SIRT1-PGC1-α signalling pathway and increases energy expenditure in adipocytes, highlighting FGF21 as one of the intermediary mechanisms between IF and AMPK activation. While PGC1-α regulates VEGF-A-mediated angiogenesis in skeletal muscle, its role in WAT remains to be determined. The mechanistic link between increased angiogenesis and WAT browning may involve endothelial cells releasing platelet-derived growth factor CC (PDGF-CC) in response to VEGF-A, leading to WAT browning. It may also be driven by alterations in immune cells, such as polarisation towards M2-like macrophages. However, the specific mechanisms remain to be elucidated. Created with BioRender.com.

The AMPK-SIRT1-PGC1α signalling axis may be a pivotal driver of VEGF-A-mediated angiogenic remodeling and browning in WAT in response to IF (Figure 2). This pathway’s relevance was illustrated in HepG2 human hepatoma cells, where AICAR-stimulated AMPK activation resulted in increased VEGF-A production [98]. Glucose starvation for 4 h in HepG2 cells, *in vitro* conditions that mimic fasting, resulted in elevated phospho-AMPK and VEGF-A mRNA expression. Furthermore, pharmacological activation of SIRT1 led to significant metabolic improvements in DIO mice, along with improved vasculature and reduced fibrosis. In 3T3-L1 preadipocytes, SIRT1 activation was associated with the increased expression of angiogenic factors, including VEGF-A [99]. This provides evidence for SIRT1 being an upstream regulator of VEGF-A. Activation of AMPK is capable of phosphorylating and stimulating PGC-1α in skeletal muscle and WAT, an essential regulator of mitochondrial biogenesis and metabolism [100, 101]. PGC-1α activation is critical for VEGF-mediated angiogenesis in skeletal muscle, although its causative role in IF-induced WAT angiogenesis has yet to be established [102]. Nonetheless, *Ppargc1a* was significantly upregulated in WAT following IF in mice and may, therefore, be an upstream regulator of WAT angiogenesis [26]. The AMPK-SIRT1- PGC1α signalling axis was, in fact, directly activated by the anti-diabetic drug Canagliflozin in adipocytes *in vitro*, with downstream effects including increased thermogenesis and energy expenditure [103]. This provides further support for the likely involvement of this pathway in IF. Although direct evidence linking IF to the activation of AMPK, SIRT1, and PGC-1α in WAT has yet to be shown, CR can activate this pathway in the heart and mitigate myocardial injury following reperfusion [104].

Fibroblast growth factor 21 (FGF21) also plays a crucial role in regulating VEGF-A expression. In mice with liver-specific knockout of FGF21 subjected to IF for 16 weeks, there was a lack of angiogenic growth and browning in WAT, as indicated by the reduced expression of browning markers such as *Ucp1*, *Ppargc1a*, and *Elovl6* [105]. Additionally, IF-mediated anti-inflammatory effects were lost following FGF21 knockout, as evidenced by an increase in pro-inflammatory M1-like macrophage markers. Since M2-like polarisation is essential for IF-induced browning, liver-derived FGF21 may serve as a critical upstream regulator in this process. Interestingly, Abu-Odeh et al highlighted that browning of iWAT following treatment with the β-adrenergic agonist CL-316,243, which mimics cold exposure, still occurred despite the liver-specific knockout of FGF21. These findings suggest that hepatic and adipocyte-derived FGF21 may have differing effects under various physiological stimuli, with liver-derived FGF21 being crucial for IF-induced browning and anti-inflammation, while adipocyte-derived FGF21 plays a key role in β-adrenergic receptor-mediated thermogenesis [106]. Nevertheless, Chau et al established that FGF21 increases energy expenditure through the phosphorylation and activation of AMPK, which in turn activates SIRT1 and PGC-1α both *in vitro* and *in vivo* [107]. Thus, IF may induce VEGF-A expression and angiogenic remodeling via an upstream pathway involving FGF21, AMPK, SIRT1, and PGC-1α. Further research is needed to confirm these mechanisms and to fully elucidate the role of FGF21 in IF-mediated angiogenesis.

The crosstalk between white adipocytes and endothelial cells is vital for angiogenesis and WAT browning. Seki et al investigated the effects of adrenergic activation induced by cold exposure and CL-316,243 treatment, which elicited a browning response similar to that observed with IF. Their research identified platelet-derived growth factor CC (PDGF-CC) as an endothelial-derived soluble factor activated by VEGF-A that promotes the differentiation of adipocyte progenitor cells towards a beige phenotype [108]. Furthermore, Seki et al demonstrated that deletion of VEGF receptor 1 (VEGFR1) in endothelial cells, a receptor that typically functions as a sink for VEGF-A and thus inhibits its function, results in WAT browning [109]. VEGF-A likely interacts with other receptors on endothelial cells besides VEGFR1, including VEGFR2 and neuropilin-2, indicating a complex network of potential signalling interactions between adipocytes and endothelial cells [110].

CR elicits a type 2 immune response similar to IF, mediating the browning of WAT [20]. Under CR conditions, SIRT1 levels were elevated in macrophages and eosinophils, a response likely driven by IL-4 [111]. In a contrasting study, J. Park et al transplanted adipose tissue overexpressing VEGF-A into DIO mice, offering a direct parallel to the IF-induced elevation of VEGF-A described by Kim et al [26, 112]. Following transplantation, angiogenic remodeling and the development of beige adipocytes were observed, independent of IL-4 [112]. Group 2 innate lymphoid cells (ILC2) have been extensively researched in adipose tissue for their role in fostering a type 2 inflammatory environment. The essential cytokines, IL-5 and IL-13 are critical for the activation of eosinophils and M2-like macrophages, thereby influencing WAT beiging and thermogenesis [113–115]. Given that VEGF-A does not directly induce the M2-like polarisation of macrophages necessary for the browning effects seen with IF, the interplay between endothelial cells, macrophages, and other immune cells, as well as proangiogenic factors will be important in determining the mechanism underlying IF-induced WAT browning.

## Intermittent fasting and sympathetic innervation

Sympathetic innervation is a key regulator of lipolysis, allowing fat mobilisation from adipose tissue in response to the body’s energy demands. In response to physiological stimuli, activation of the SNS drives the release of norepinephrine (NE) from local sympathetic nerves into the adipocyte microenvironment. This subsequently activates β-adrenergic G-protein coupled receptors expressed on adipocytes, including ADRB1, ADRB2, and ADRB3*,* resulting in the decoupling of the G_S_ protein and activation of adenylate cyclase (AC). This results in an increase in levels of intracellular cAMP, which activates protein kinase A (PKA) and culminates in a signalling cascade that phosphorylates key lipolytic enzymes such as HSL and perilipin A (PLIN1a) [116]. Activated HSL translocates to the lipid droplet monolayer, and ATGL is subsequently activated by the release of ABHD5 from activated PLIN1a, catalysing the hydrolysis of triglycerides [117].

Early evidence from Migliorini et al indicated increased sympathetic activity in epididymal WAT after 48 h of fasting, as evidenced by an increased NE turnover rate [118]. Additionally, 48-hour fasting has also been shown to stimulate sympathetic innervation, as evidenced by increased tyrosine hydroxylase (TH) protein content, in both epididymal and retroperitoneal WAT [119]. β-adrenergic activity can alter the expression of GLUT4 in WAT in response to short-term fasting and refeeding, thereby regulating glucose homeostasis [120]. It remains unclear whether IF utilises a similar mechanism to improve glucose homeostasis. IF also increases lipolysis through elevated phosphorylation of HSL and extracellular signal-regulated kinase (ERK), a kinase that activates lipolytic enzymes, including HSL [44]. The upregulation of *Adrb3* expression in mouse WAT under IF suggests that sympathetic activation of adrenergic receptors may initiate the downstream lipolytic cascade [26]. A similar mechanism is observed in humans, where propranolol-stimulated blockade of β-adrenergic receptors can mitigate fasting-induced lipolysis [121].

In contrast, Li et al elucidated an opposite effect of IF leading to reduced *Adrb3* expression in both BAT and WAT. Their study highlighted the importance of gut microbiota changes in driving WAT browning after every-other-day feeding (EODF), a regimen synonymous with ADF [49]. A separate study using proteomic analysis on fat pads from EODF-treated mice revealed a reduction in ADRB3 and lipolytic pathways, including reduced monoacylglycerol lipase, along with an upregulation in fatty acid synthesis pathways involving enzymes such as ATP-citrate lyase. This suggests a potential mechanism for energy conservation [122]. Given the evident reduction in calorie intake associated with EODF, the observed attenuation of lipolysis may be a metabolic adaptation compared to the isocaloric nature of the 2:1 IF regimen, which activates ADRB3 [26]. These findings underscore the importance of the specific type of IF intervention on WAT remodeling and its downstream functional effects.

**FIGURE 3:**
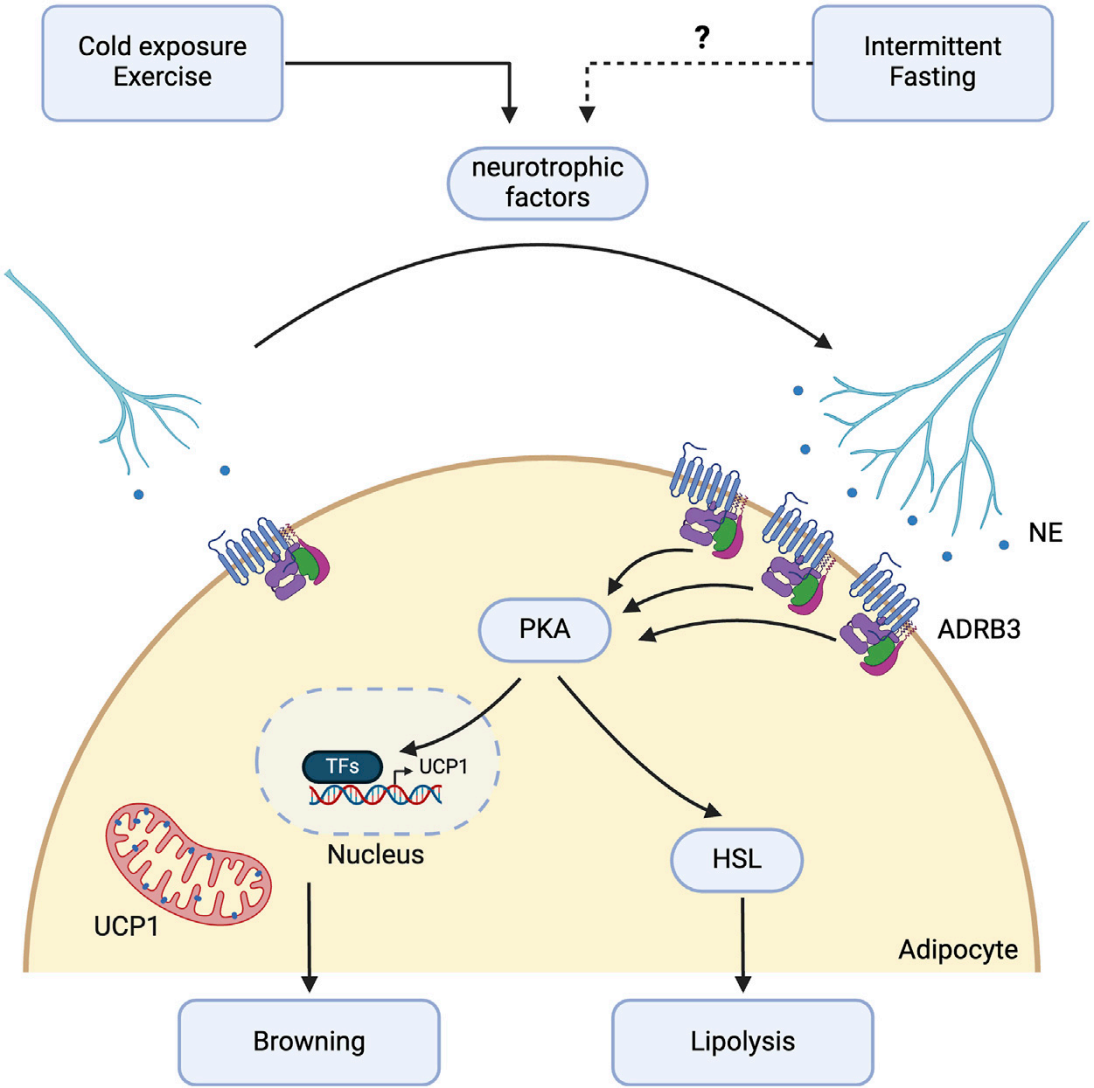
Potential molecular pathways involved in intermittent fasting-induced sympathetic innervation. Intermittent fasting (IF) may promote sympathetic activation in white adipose tissue (WAT) through the β-3 adrenergic signalling pathway. Although direct evidence showing IF’s effect on sympathetic nerve growth in WAT is currently lacking, existing studies suggest that IF may promote this growth through VEGF-A or other neurotrophic factors such as neuronal growth regulator 1 (NEGR1), neurotrophin-3 (NT-3), neuregulin 4 (NRG4), nerve growth factor (NGF), and slit guidance ligand 3 (SLIT3), which are implicated in exercise or cold-induced sympathetic nerve growth. IF enhances β-3 adrenergic receptor expression and downstream lipolysis through activation of hormone-sensitive lipase (HSL). Sympathetic innervation further promotes the browning of WAT via the protein kinase A (PKA) intracellular pathway, increasing the expression of thermogenic genes such as uncoupling protein 1 (UCP1). Created with BioRender.com.

The molecular mechanisms by which IF results in adaptive remodeling of the neural architecture of WAT, thereby promoting lipolysis and thermogenesis, remain to be investigated (Figure 3). Current evidence suggests that IF facilitates angiogenesis via upregulation of VEGF-A [26]. Separately, Zhao et al showed that VEGF-A overexpression in transgenic mice not only increases angiogenesis but also promotes sympathetic nerve growth [123]. VEGF-A overexpression leads to downstream activation of ADRB3 and phosphorylation of HSL, as well as increased expression of browning markers including UCP1 and PGC1- α*.* This highlights a potential mechanism by which IF may mediate lipolysis and browning via VEGF-A-induced sympathetic nerve growth [123].

Stimuli, such as exercise and cold exposure, which activate ADRB3 and confer metabolic benefits similar to those of IF, have been observed to stimulate both sympathetic innervation and angiogenesis in WAT. In addition to VEGF-A, several neurotrophic factors released in WAT regulate this remodeling process. Neuronal growth regulator 1 (NEGR1), a cell adhesion molecule that modulates neural innervation in the brain, is significantly associated with body mass index (BMI) in meta-analyses of genome-wide association studies [124, 125]. Exercise training for 10 weeks in C57BL/6 mice induced NEGR1 expression specifically from mature white and beige adipocytes in iWAT, leading to increased neurite growth. The increase in NEGR1 expression has also been observed in humans following treadmill training, with PRDM16, regulated by PPARγ, identified as the critical transcription factor governing NEGR1 expression [126].

Neurotrophin-3 (NT-3) and neuregulin 4 (NRG4) are adipocyte-derived growth factors that mediate sympathetic nerve growth in WAT and promote beige fat formation under cold conditions [8, 10]. NT-3 binds to TrKC receptors in the sympathetic ganglia, while NRG4 functions through ErbB4 [127]. NRG4 can also directly stimulate browning in adipocytes *in vitro* [128]. Cold-induced production of nerve growth factor (NGF) by adipose eosinophils also stimulates neurite growth [129]. NGF, recognised as an adipokine, targets TrkA receptors in iWAT and is necessary for cold-stimulated beiging [130]. Increased NGF production is attributed to an increased accumulation of adipose eosinophils, driven by increased IL-5 secretion from ILC2s, which in turn is stimulated by IL-33 release from stromal cells in response to cold. Cold-induced sympathetic nerve growth is also mediated by the secretion of slit guidance ligand 3 (SLIT3) by M2-like macrophages, which bind to roundabout guidance receptor 1 (ROBO1) receptors on nerve fibres. The SLIT3-ROBO1 interaction promotes increased NE release, leading to the upregulation of the lipolytic and thermogenic pathways, marked by elevated PKA activity, phosphorylation of HSL, and increased UCP1 expression [131].

A plethora of neurotrophic factors regulate sympathetic nerve growth in WAT in response to exercise and cold exposure. However, whether these factors play a specific role in mediating IF-stimulated adipose tissue remodeling remains to be elucidated. IF is known to shift the immune cell landscape towards a type 2 inflammatory response with increased M2-like macrophage polarisation and eosinophils, as observed in aged mice subjected to IF [132]. This contrasts with the pro-inflammatory environment in obese adipose tissue, characterised by reduced ILC2 and eosinophil populations and a shift towards M1-like macrophages [133–135]. These immune alterations may contribute to the underlying mechanisms leading to reduced sympathetic innervation of WAT in obesity [136]. Furthermore, whether AMPK plays a role in regulating SNS activation remains uncertain. Nevertheless, AMPK is known to directly regulate lipolysis through the phosphorylation of ATGL and HSL [137]. Additionally, the process of lipolysis itself leads to an increase in the AMP:ATP ratio, which may result in the activation of AMPK [138].

## Discussion

Recent studies have established IF as an effective, economical, and practical strategy for weight management. Meta-analyses and umbrella reviews conducted in recent years highlight the efficacy of IF as a dietary intervention in improving health outcomes in obese or overweight participants, including reduced body weight and fat mass, favourable lipid profiles, reduced inflammatory markers, and improved fasting insulin as well as plasma glucose levels [139–143]. Beyond its efficacy in obese adult populations, IF may reduce BMI and the risk of cardiovascular disease in adolescents with obesity [144]. Despite the known metabolic health advantages associated with IF, compliance with a dietary regimen like IF can pose a challenge in the long term. However, adherence and feasibility can be maintained by implementing a personalised dietary regimen under the guidance of a professional dietitian and by building flexibility into the programme, allowing users to choose specific days of the week to fast or restrict calories [144].

To mediate the beneficial effects of IF on WAT remodeling and systemic metabolic homeostasis, IF may modulate a complex regulatory network involving signalling pathways such as PI3K/Akt, SIRTs, and mTOR. As AMPK is positioned as the central metabolic switch regulating the downstream pathways, activation of AMPK in response to IF may be integral in promoting a range of cellular processes, including lipolysis, β-oxidation, autophagy, and the browning of WAT. Nevertheless, both gain-of-function and loss-of-function studies of these intracellular pathways, especially in WAT, are necessary to validate their relevance in IF-mediated WAT remodeling. VEGF-A drives IF-induced angiogenesis in WAT, which may, in part, be regulated by the AMPK-SIRT1-PGC1α signalling axis. FGF21 has also emerged as an essential signalling factor upregulated by IF, which can activate AMPK and downstream PGC-1α [105]. VEGF-A, along with other neurotrophic factors, can promote sympathetic nerve growth, highlighting the close interplay observed in the remodeling of the neurovascular architecture of WAT. While IF can promote angiogenesis and sympathetic activation [26], its direct effect on sympathetic nerve growth has not been directly elucidated, with current evidence derived from studies on exercise [126] and cold-induced sympathetic nerve growth [8, 10, 130, 131] in WAT.

Additionally, the current mechanistic understanding of IF remains limited, particularly regarding its direct activation of pathways such as AMPK, PI3K/Akt, various SIRTs, and mTOR in WAT. The majority of the available evidence comes from studies in CR or from other tissues such as the heart [58, 59, 61, 80, 81, 83, 97], liver [56, 57], and brain [62, 63]. Furthermore, the variability between different IF regimens [26, 28, 49, 122] necessitates further investigation into their physiological effects and underlying mechanisms, which is crucial for standardising IF protocols for research and clinical applications. These differences also underscore the importance of thoroughly investigating both the benefits and downsides of IF. For example, ADF has been shown to exacerbate atherosclerosis in mice predisposed to the condition, and TRF increases hepatic insulin resistance in young rats with diet-induced obesity [145, 146]. Understanding these differences and potential adverse effects is essential for identifying appropriate target populations for IF.

It is also essential to consider the cyclical nature of IF with its fasting and refeeding cycles. The periodic activation and inhibition of these signalling pathways may be important in the development and administration of pharmacological mimetics. Intermittent administration of rapamycin can prolong the life of female C57BL/6 mice while negating any metabolic side effects such as impaired insulin sensitivity [147]. Similarly, periodic administration of metformin, an AMPK activator, showed metabolic improvements and weight loss without affecting the mortality of aged mice, despite its toxic side effects at the dosage used [148].

AMPK activators have demonstrated potential for promoting weight loss through the activation of AMPK, leading to the inhibition of fat synthesis and promotion of fat oxidation pathways. However, their clinical use has been predominantly focused on diabetes management rather than weight loss, possibly due to the wide range of targets affected by AMPK activators like metformin [149, 150]. Further research is needed to determine their specific mechanisms of action. The activity of AMPK is also highly dependent on several factors, such as intracellular ATP levels, dosage, and route of administration [151–153]. Additional studies are required to investigate whether AMPK’s functional role in fat metabolism can be separated from its role in glucose regulation, thus supporting its potential as a standalone target for weight loss [154]. Several side effects associated with the use of AMPK activators, including gastrointestinal discomfort, headaches, and fatigue, may compromise adherence, effectiveness, and patient quality of life [153, 155].

In conclusion, IF holds great promise as a dietary intervention for weight loss and the improvement of metabolic health through its multifaceted effects on WAT remodeling. Addressing the gaps in our understanding of the molecular mechanisms driving these processes will enable the development of more targeted drug therapies that can replicate or even enhance the metabolic benefits of IF, potentially circumventing the need for significant dietary changes. Future studies should focus on standardising IF protocols, investigating the interplay between key signalling pathways, and exploring the potential of pharmacological mimetics or a combination of IF and pharmacotherapy to provide a comprehensive approach to obesity management.

## Figures and Tables

**Table:** 

**T2D**	Type 2 Diabetes
**WAT**	White Adipose Tissue
**ASPCs**	Adipose Stem and Progenitor Cells
**CR**	Calorie Restriction
**IF**	Intermittent Fasting
**ADF**	Alternate-Day Fasting
**PF**	Periodic Fasting
**TRF**	Time-Restricted Feeding
**RF**	Ramadan Fasting
**UCP1**	Uncoupling Protein 1
**PI3K**	Phosphatidylinositol 3-Kinase
**Akt**	Protein Kinase B
**CLK2**	CDC-like kinase 2
**AMPK**	AMP-activated protein kinase
**DIO**	Diet-Induced Obesity
**SIRTs**	Sirtuins
**NAD+**	Nicotinamide adenine dinucleotide
**HFD**	High-Fat Diet
**mTOR**	Mammalian Target of Rapamycin
**mTORC1**	mTOR Complex 1
**mTORC2**	mTOR Complex 2
**ATGL**	Adipose Triglyceride Lipase
**HSL**	Hormone-Sensitive Lipase
**TFEB**	Transcription Factor EB
**VEGF**	Vascular Endothelial Growth Factor
**VEGF-A**	Vascular Endothelial Growth Factor A
**VEGFR2**	Vascular Endothelial Growth Factor Receptor 2
**FOXO1**	Forkhead Box O1
**GSK3β**	Glycogen Synthase Kinase 3 Beta
**PPARγ**	Peroxisome Proliferator-Activated Receptor Gamma
**SREBP1-c**	Sterol Regulatory Element-Binding Protein 1-c
**AMP**	Adenosine monophosphate
**ATP**	Adenosine triphosphate
**ULK1**	UNC-51-like kinase 1
**pWAT**	Perigonadal White Adipose Tissue
**iWAT**	Inguinal White Adipose Tissue
**PGC-1α**	Peroxisome Proliferator-Activated Receptor Gamma Coactivator 1-Alpha
**FGF21**	Fibroblast Growth Factor 21
**PDGF-CC**	Platelet-Derived Growth Factor CC
**VEGFR1**	Vascular Endothelial Growth Factor Receptor 1
**IL-4**	Interleukin 4
**ILC2**	Group 2 Innate Lymphoid Cells
**NE**	Norepinephrine
**ADRB1**	Adrenergic Receptor Beta 1
**ADRB2**	Adrenergic Receptor Beta 2
**ADRB3**	Adrenergic Receptor Beta 3
**AC**	Adenylate Cyclase
**PKA**	Protein Kinase A
**PLIN1a**	Perilipin A
**TH**	Tyrosine Hydroxylase
**ERK**	Extracellular Signal-Regulated Kinase
**GLUT4**	Glucose Transporter Type 4
**EODF**	Every other day feeding
**BMI**	Body Mass Index
**NEGR1**	Neuronal Growth Regulator 1
**NT-3**	Neurotrophin-3
**NRG4**	Neuregulin-4
**ErbB4**	Erb-B2 Receptor Tyrosine Kinase 4
**TrkC**	Tropomyosin Receptor Kinase C
**NGF**	Nerve Growth Factor
**TrkC**	Tropomyosin Receptor Kinase A
**SLIT3**	Slit Guidance Ligand 3
**ROBO1**	Roundabout Guidance Receptor 1
